# Extended duration of the detectable stage by adding HPV test in cervical cancer screening

**DOI:** 10.1038/sj.bjc.6601355

**Published:** 2003-11-11

**Authors:** M E van den Akker-van Marie, M van Ballegooijen, L Rozendaal, C J L M Meijer, J D F Habbema

**Affiliations:** 1 Department of Public Health, Erasmus MC, PO Box 1738, 3000 DR Rotterdam, The Netherlands; 2 Department of Pathology, VU MC, Amsterdam, The Netherlands

**Keywords:** human papillomavirus, cervical cancer screening model

## Abstract

The human papillomavirus test (HPV) test could improve the (cost−) effectiveness of cervical screening by selecting women with a very low risk for cervical cancer during a long period. An analysis of a longitudinal study suggests that women with a negative Pap smear and a negative HPV test have a strongly reduced risk of developing cervical abnormalities in the years following the test, and that HPV testing lengthens the detectable stage by 2–5 years, compared to Pap smear detection alone.

One of the possible uses of the human papillomavirus test (HPV) is in primary cervical cancer screening in addition to or instead of the current Pap smear (Cuzick *et al*, 1999a; Cuzick, 2000; Meijer and Walboomers, 2000).

Introduction of HPV screening should be based on established (cost-) effectiveness. The (cost-) effectiveness of HPV testing is primarily determined by the duration of the detectable preclinical stage (the period from the HPV infection to clinical disease), and the sensitivity and costs of HPV testing. To estimate preclinical duration and sensitivity, longitudinal studies on the association between HPV infection and the development of neoplasias are necessary. Several large longitudinal screening studies have started, but no longterm results have been reported yet, although smaller longitudinal studies have been published (Rozendaal *et al*, 1996, 2000; Ho *et al*, 1998; Liaw *et al*, 1999). These studies differ with respect to HPV test used, age range of women, study design, and cytological or histological endpoint, which complicates the comparison and interpretation of these results. It is therefore too early for definite answers on the value of HPV testing in primary screening (Myers *et al*, 2000; Kim *et al*, 2002; Mandelblatt *et al*, 2002). However, available data can be explored to derive preliminary estimates for parameters that determine the cost-effectiveness of HPV testing. This study investigates the duration of the detectable preclinical stage using the results of Rozendaal *et al* (2000). For these estimates, the 5-year cumulative incidence of cervical intraepithelial neoplasia (CIN) III after a negative Pap smear, the current screening interval in the Netherlands, is compared with the cumulative incidence within a doubled screening interval of 10 years (Meijer and Walboomers, 2000) in case of a negative Pap smear and a negative HPV test.

## MATERIAL AND METHODS

The study population, and screening and follow-up results are described in Rozendaal *et al* (1996), (2000). Briefly, the smears obtained during routine screening from 1988 to 1991, from a cohort of 2250 women aged 34–54 years, who were either normal or who showed borderline nuclear changes were tested for a high-risk HPV. The women were followed during a mean period of 6.4 years, using screen-detected (histologically confirmed) CIN III as end point. Among the 2129 (95%) women with a negative HPV test at baseline, one case of CIN III was diagnosed at a following screening round. Of the 121 women with a positive HPV test result at baseline (5%), 12 women with CIN III were detected later. This resulted in a relative risk of 210, with a 95% confidence interval from 27 to 1600.

**Figure 1:**
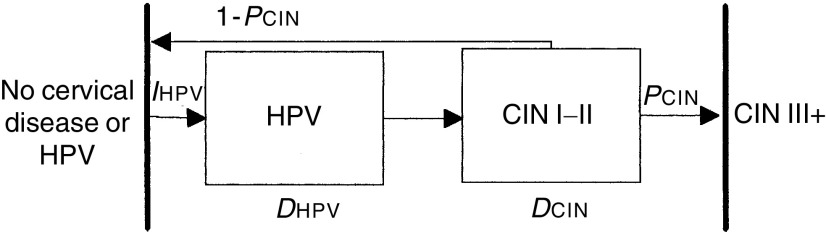
Schematic representation of the natural history model for HPV and CIN. *I*_HPV_=incidence rate of HPV infections in women without cervical disease or HPV, *P*_CIN_=probability that HPV will progress to CIN III, *D*_HPV_=duration of HPV infection preceding CIN and *D*_CIN_=duration of CIN I/II preceding CIN III.

The disease model used in this study is schematically presented in Figure 1

. Women without cervical disease or HPV may become infected with HPV. This infection may clear or it may progress to low-grade CIN. From ‘low-grade CIN’, the disease may regress spontaneously or progress to high-grade CIN (corresponding with CIN III), the end point of the model. We assume that CIN III cannot develop without HPV infection.

In the model, we assumed a constant duration of the HPV infection, and an exponentially distributed duration of low-grade CIN with a mean of 6 years. The incidence rate of HPV infections in the age group considered (34–54 years) was set at five per 1000 woman years (Meijer, 1997). Using the results of the Rozendaal study, the duration of the HPV infection and the probability that the HPV infection will progress to CIN III were estimated. On the basis of these estimates, it was possible to calculate the cumulative incidence of CIN III within 5 years after the smear was taken; the current screening interval in the Netherlands, per 1000 cytologically negative women and the cumulative incidence of CIN III within 10 years after the smear was taken per 1000 cytologically negative/HPV negative women.

Initially, it was assumed that there were no diagnostic errors, that is, the results of the HPV test and the Pap smear as found by Rozendaal *et al* (2000) reflected the true disease stage of the women. We used this as our reference model. In alternative models, we studied the consequences of assuming diagnostic errors. We also varied the assumptions on the incidence rate of HPV infections and the duration of low-grade CIN. The mathematical description of the model is given in the Appendix.

## RESULTS

In Table 1

**Table 1:** Estimated values for the duration of HPV, the probability that the HPV infection will progress to CIN III, the 5-year cumulative incidence in women with a negative smear and the 10-year cumulative incidence in women with a negative smear and a negative HPV test

	**Duration HPV (years)**	**Probability HPV progresses to CIN III**	**5-year cum. incidence CIN III after cyt −(per 1000 women)**	**10-year cum. incidence CIN III after cyt−/HPV-(per 1000 women)**
Reference model^a^	3.8	0.19	4.1	2.2
Sensitivity HPV test for HPV 50%^b^	2.2	0.08	4.6	5.2
Sensitivity cytology for CIN I+ 50%	3.5	0.16	10.0c	4.0^c^
Incidence HPV infections 0.0025	2.3	0.17	4.5	1.4
Incidence HPV infections 0.010	4.7	0.20	3.8	3.7
Mean duration CIN I/II 2 years	4.3	0.11	4.5	2.2
Mean duration CIN I/II 10 years	3.6	0.27	4.0	2.2

a Reference model: mean duration CIN I/II 6 years, sensitivity HPV test for HPVinfection 100%, sensitivity cytology for CIN I+100% and incidence HPV infections 0.005 per woman year.

b Assuming that the one woman that developed CIN III in (Rozendaal *et al*, 2000) after HPV negative test at baseline did not have a false-negative HPV test result.

c Owing a limited sensitivity of cytology for CIN III, only part of these lesions will be detected within the follow-up period considered.

, the results of the reference and alternative models are shown. Using the reference values for the model parameters, the duration of HPV infection before progressing to CIN was estimated at 3.8 years, resulting in a lower cumulative incidence of CIN III in 10 years for women with double-negative screening results, than in 5 years after a negative Pap smear and an unknown HPV result.

Next, we dropped the perfect-test assumptions (100% sensitivity) of the HPV test and Pap smear separately, by assuming 50% sensitivity for detecting an HPV infection and 50% sensitivity for detecting CIN I+, respectively. Furthermore, we halved and doubled our assumptions on the incidence rate of HPV infections and the duration of low-grade CIN. The estimated range for the duration of HPV before progressing to CIN widened, from 2 to 5 years. Only where the HPV test was assumed to have a sensitivity of 50% for HPV infections that will progress to CIN does the cumulative incidence 10 years after a double-negative result become slightly higher than the incidence within 5 years after a negative Pap smear.

## DISCUSSION

Our analysis shows duration of the HPV infection before it will progress into CIN of 2–5 years. For Pap smear screening, the preclinical duration was the combined duration of CIN and microinvasive cervical cancer, a period estimated at 15 years on average (Gustafsson and Adami, 1989; van Oortmarssen and Habbema, 1991, 1995; van Oortmarssen *et al*, 1992; Zielinski *et al*, 2001). Consequently, adding the HPV test to primary screening leads to duration of the detectable preclinical stage of almost 20 years in women aged 34–54 years. Furthermore, the 10-year cumulative incidence in women with a negative Pap smear and HPV test was lower than the 5-year cumulative incidence in women with a negative Pap smear and an unknown HPV result. This high negative predictive value of CIN III in double-negative women is the result of a longer preclinical duration and a better selection of women, as women with double-negative test results are at a lower risk of cervical cancer than women with only negative Pap smear results of whom part will have HPV infections.

These results suggest that an HPV test, in combination with the Pap smear, can considerably lengthen the screening interval in double-negative women (Meijer and Walboomers, 2000).

The confidence interval around the relative risk found by Rozendaal *et al* (1996) was large (from 27 to 1600). The upperbound of the confidence interval results in an HPV infection duration of 5.5 years. Assuming a relative risk corresponding to the lowerbound, results in a negligible duration. Even then, the 10-year cumulative incidence in double-negative women is lower than the 5-year cumulative incidence in women with a negative cytological result, assuming no diagnostic errors. This results from the very low risk of double-negative women of becoming infected with HPV infected, and subsequently developing cervical abnormalities in the years following the test. More firm estimates will be obtained on the basis of the results of the ongoing longitudinal studies.

The relative risks found in other studies (Ho *et al*, 1998; Liaw *et al*, 1999), 10.0 and 12.7, respectively, are lower than the range studied here. One of the reasons may be that the other studies concern women aged around 20 years. In young women, the occurrence of HPV infections is high (Melkert *et al*, 1993) and a much higher proportion of these infections are transient (Evander *et al*, 1995) compared to older women. Therefore, adding the HPV test in primary screening is not useful for young women (Cuzick *et al*, 1999b).

With the current model, it is technically not possible to lower the sensitivity of the HPV test and Pap smear simultaneously. Doing this will probably result in an estimate for the duration of the HPV infection of around 2 years. Also, the assumption of a constant duration of the HPV infection can be dropped using a more sophisticated model. However, these refinements pay off only when adequate longitudinal data on HPV detection are available for quantification of the additional parameters.

Women may develop CIN III without first passing the stages CIN I (and even CIN II). This situation has been represented by assuming a relatively short average duration of low-grade CIN of 2 years. Together with the assumption that this stage is exponentially distributed among women, this leads to a situation in which part of the women will develop CIN III shortly after having no neoplasia. Under these assumptions, the duration of the HPV infection before progressing to CIN is estimated to be relatively long, and the selection of low-risk women by adding HPV to cytology will be even better (incidence of CIN III 2.2 *vs* 4.5, Table 1).

The end point of the model was CIN III as imposed by the data. Invasive cancer is the end point to be preferred as prevention of invasive cancer and therefore, death is aimed at by cervical cancer screening. This end point, however, does not yield sufficient power due to the low risk for invasive cancer in Pap smear screened women, unless extremely large and long-term trials are performed. The current estimate on the duration of HPV before developing CIN is a combined estimate for the duration of HPV for women who will have a regressive CIN III lesion and those who will progress to cervical cancer. To solve the uncertainty on the confounding of regressive CIN III lesions, this prospective analysis with CIN III as end point should be accompanied by archival studies, in which retrospectively the HPV status of smears preceding a diagnosis of cervical cancer, is assessed. Zielinski *et al* (2001) concluded in a retrospective study of 57 women with invasive cervical cancer that the detectable preclinical stage could be prolonged by at least 2 years by adding HPV testing, which corroborates our results. This type of study, however, is susceptible to confounding biases such as selection and length time bias, which may result in an underestimation of the extension of the detectable preclinical stage as cervical cancers found after participation in a screening programme may be selective towards fast-growing cancers.

Doubling the screening interval for double-negative women will result in cost savings, as half of the screening rounds can be omitted. If, for example, the effectiveness of 10-yearly combined screening is the same as 5-yearly screening using the Pap smear, the costs of adding the HPV test to the Pap smear must be lower than these savings to be at least a cost-equal alternative. For a full cost analysis, other costs and savings should also be taken into account, such as the costs of follow-up in HPV positive/cytologically negative women, and possible savings due to a decrease in the detection of regressive cervical lesions because of a longer screening interval.

In conclusion, adding the HPV test to cytology in primary screening for cervical cancer results in an additional duration of the detectable preclinical stage of 2–5 years. Consequently, the screening interval for women with cytological and HPV-negative test results may be considerably lengthened. These results remain to be confirmed by the large longitudinal studies that are currently underway.

## Appendix

Mathematical formulae of the reference model

If we assume that:

- incidence rate of HPV is *I*_HPV_,
- probability that HPV will progress to CIN III is *P*_CIN_,
- duration of HPV preceding CIN is *D*_HPV_ and
- duration CIN I/II preceding CIN III is exponentially distributed with mean *D*_CIN_.

Then, cumulative incidence of CIN III after *x* years for women without cervical lesions and without HPV infection at baseline

Cumulative incidence of CIN III after *x* years for women without cervical lesions but with HPV infection at baseline

Relative risk on CIN III in women not having a cervical lesion but being HPV infected compared to women without cervical lesion and HPV infection after *x* years

Cumulative incidence of CIN III *x* years after not having a cervical lesion

with *p*_HPV−_ and *p*_HPV+_ the percentage of women without and with HPV infection at baseline, respectively.
